# 

**Published:** 1937

**Authors:** 


### CONTENTS.

*Page*

Nutrition and Tuberculosis. By Prof. J. A. Nixon, C.M.G., M.D., F.R.C.P. .. .. .. .. .. .. .. .. 191

The Burden Mental Research Trust: its Present and Future. By R. J. A. Berry, M.D., F.R.S.E. .. .. .. .. 201

Sulphanilamide in Treatment. By E. Watson-Williams, M.C., Ch.M., F.R.C.S. .. .. .. .. .. .. .. 209

Prontosil in Obstetrics. By H. J. Drew-Smythe, M.S., F.R.C.S., F.C.O.B. .. .. .. .. .. .. .. .. 217

Ham Green Hospital Extensions. The Opening Address delivered by Professor W. W. Jameson, M.D., F.R.C.P., D.P.H., Barrister-at-Law .. .. .. .. .. .. .. .. 221

Reviews of Books .. .. .. .. .. .. .. .. 227

Editorial Notes .. .. .. .. .. .. .. .. 231

The Medical Library of the University of Bristol .. .. 234

Publications Received .. .. .. .. .. .. .. .. 236

Local Medical Notes .. .. .. .. .. .. .. 237

